# On the Use of 4D-PET/CT for the Safe SBRT Re-Irradiation of Central Lung Recurrence Within Radiation-Induced Fibrosis: A Clinical Case

**DOI:** 10.3390/jcm14124015

**Published:** 2025-06-06

**Authors:** Paul Retif, Emilie Verrecchia-Ramos, Motchy Saleh, Abdourahamane Djibo Sidikou, Romain Letellier, Anwar Al Salah, Estelle Pfletschinger, Fabian Taesch, Sinan Ben-Mahmoud, Xavier Michel

**Affiliations:** 1 Medical Physics Unit, Centre Hospitalier Régional Metz-Thionville, 57000 Metz, France; emilie.verrecchia@chr-metz-thionville.fr (E.V.-R.); motchy.saleh@chr-metz-thionville.fr (M.S.); abdourahamane.djibo-sidikou@chr-metz-thionville.fr (A.D.S.); romain.letellier@chr-metz-thionville.fr (R.L.); anwar.alsalah@chr-metz-thionville.fr (A.A.S.); estelle.pfletschinger@chr-metz-thionville.fr (E.P.); fabian.taesch@chr-metz-thionville.fr (F.T.); 2 Université de Lorraine, Centre National de la Recherche Scientifique (CNRS), Centre de Recherche en Automatique de Nancy (CRAN), 54000 Nancy, France; 3 Nuclear Medicine Department, Centre Hospitalier Régional Metz-Thionville, 57000 Metz, France; sinan.ben-mahmoud@chr-metz-thionville.fr; 4 Radiation Therapy Department, Centre Hospitalier Régional Metz-Thionville, 57000 Metz, France; xavier.michel@chr-metz-thionville.fr

**Keywords:** re-irradiation, 4D-PET/CT, central lung SBRT

## Abstract

**Background:** The re-irradiation of centrally located lung tumors poses substantial risks due to prior dose exposure and proximity to critical structures. Accurate target delineation is crucial to minimize toxicity and ensure tumor coverage. Four-dimensional positron emission tomography/computed tomography (4D-PET/CT) integrates respiratory motion and metabolic data, offering improved delineation over static imaging. Its clinical utility in re-irradiation remains under-reported. **Methods:** A 67-year-old male presented with the central recurrence of squamous cell carcinoma in the right upper lobe, embedded in radiation-induced fibrosis, following prior chemoradiotherapy. Delineation using static PET underestimated tumor motion. A 4D-PET/CT-guided Stereotactic Body Radiation Therapy (SBRT) plan was developed with a prescription of 60 Gy in eight fractions. A comparative plan using static PET was generated to assess the dosimetric differences. **Results:** The internal target volume (ITV) from 4D-PET/CT was nearly double the size of the GTV from static PET, with a 5.1 mm discrepancy in the craniocaudal axis. The 4D-PET-based plan achieved 95.0% PTV coverage, while the static PET-based plan covered only 61.7%, illustrating the risk of underdosage without motion-resolved imaging. The patient completed the treatment without acute or late toxicity and showed a sustained metabolic response at one year (SUVmax from 13.4 to 5.8). **Conclusions:** This case demonstrates the clinical value of 4D-PET/CT in the SBRT re-irradiation of centrally located lung tumors, particularly in fibrotic regions where anatomical imaging is insufficient. It enabled accurate delineation, improved dosimetric coverage, and safe, effective retreatment. These findings support its integration into planning for complex thoracic re-irradiation.

## 1. Introduction

Stereotactic Body Radiation Therapy (SBRT) has become a standard treatment modality for patients with inoperable early-stage non-small-cell lung cancer, offering high rates of local control with a favorable toxicity profile [1,2,3,4]. Owing to its high spatial precision, SBRT allows for the significant reduction in Planning Target Volume (PTV) margins, thereby enabling dose escalation without increased toxicities, particularly for peripheral lung tumors [5,6,7].

However, the application of SBRT to centrally located lung tumors, defined by their proximity to critical structures, such as the main bronchi, the great vessels, and the esophagus, remains particularly challenging. These tumors are associated with a higher risk of severe toxicity, including fatal airway necrosis and hemorrhage, and thus require meticulous planning and stringent motion management [8,9]. This complexity is further amplified in the context of thoracic re-irradiation, where previously irradiated tissues exhibit an altered anatomy and increased susceptibility to toxicity.

Accurate target delineation is paramount in thoracic SBRT to ensure optimal tumor coverage, while minimizing the dose to the surrounding Organs At Risk (OARs) [10]. Respiratory-induced tumor motion remains a major source of uncertainty in treatment planning and delivery, and several motion management techniques have been developed to address this, including Four-Dimensional Computed Tomography (4D-CT) [11,12]. While 4D-CT provides anatomical information across respiratory phases, it does not offer metabolic characterization, which may be particularly important when tumors are located within post-radiation fibrosis or atelectasis.

In this regard, Four-Dimensional Positron Emission Tomography/Computed Tomography (4D-PET/CT) has emerged as a powerful imaging modality, combining both the temporal and metabolic resolutions. It enables the more accurate delineation of metabolically active tumor volumes, while accounting for respiratory motion [13,14,15,16]. This is especially critical in post-radiation settings, where distinguishing recurrent tumor from fibrotic tissue is challenging using CT alone [17,18,19,20,21,22,23,24]. Several dosimetric studies have demonstrated that 4D-PET/CT improves target definition, but clinical illustrations in high-risk settings remain limited [13,14,15,16,24,25].

This short communication presents a clinical case of central lung tumor recurrence located within extensive radiation-induced fibrosis, for which SBRT re-irradiation was performed using motion-compensated 4D-PET/CT for target delineation. Through this case, we aim to demonstrate the practical clinical impact of 4D-PET/CT in enabling safe and effective high-dose retreatment in anatomically and dosimetrically complex thoracic re-irradiation scenarios. We also discuss the institutional workflow and the safety framework that supported the decision-making process.

## 2. Detailed Case Description

### 2.1. Patient Description and Case History

**Figure 1:**
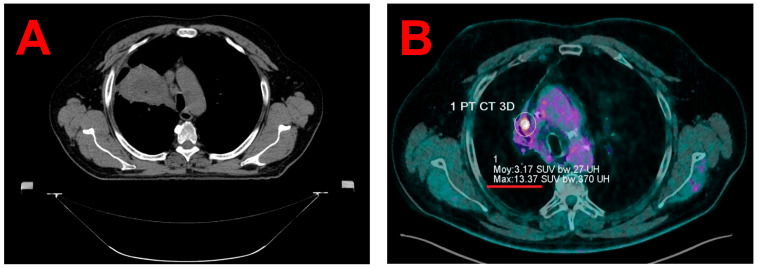
An axial view from planning CT of the initial 2020 radiotherapy treatment, showing the extensive infiltration of squamous cell carcinoma in the right upper lobe, (**A**) and an axial view from 7 December 2023 follow-up PET/CT, showing the progression of a residual para-mediastinal hypermetabolic focus located within a region of extensive radiation-induced fibrosis in the right upper lobe (SUVmax 13.4 vs. 4.7 in January 2023) (**B**).

Written informed consent was obtained from the patient for both treatment and the publication of this case report, including the associated images. The patient is a 67-year-old male (height 1.70 m; weight 108 kg) initially diagnosed in 2020 with squamous cell carcinoma of the right upper lobe. He received concurrent chemoradiotherapy (Carboplatin–Paclitaxel, 4 cycles) with a total dose of 66 Gy delivered in 33 fractions (Figure 1A), followed by adjuvant immunotherapy with Durvalumab (AstraZeneca, Wilmington, DE, USA) for two years. During follow-up, a PET/CT scan on 23 October 2023 revealed the progression of a hypermetabolic lesion in the right upper lobe, with a maximum Standardized Uptake Value (SUVmax) of 9.8 compared to 4.7 on a prior scan in January 2023. Local recurrence was suspected. This case was presented at a multidisciplinary tumor board on 2 November 2023, which recommended SBRT and the initiation of Nivolumab. Follow-up PET/CT on 7 December 2023 confirmed further metabolic progression, with an increased SUVmax of 13.4 (Figure 1B), and the lesion was noted to be embedded in a region of dense post-radiation fibrosis.

### 2.2. Planning Image Acquisitions

For radiotherapy planning, a 4D-CT scan was acquired using a Canon Aquilion LB CT scanner (Canon Medical Systems Corporation, Ōtawara, Japan). Respiratory motion was recorded with the Respiratory Gating for SCanners (RGSC) system (Varian, a Siemens Healthineers company, Palo Alto, CA, USA). The acquisitions were reconstructed into 10 respiratory-phase subsets with 2 mm slice thickness and exported to the Varian Eclipse 16.1 Treatment Planning System (TPS) (Varian, a Siemens Healthineers company, Palo Alto, CA, USA), where the Average Intensity Projection (AIP) was generated.

Given that the recurrence was located within a region of extensive fibrosis, the accurate delineation of planning 4D-CT alone proved challenging (see Figure 1B). Consequently, a planning 4D-PET/CT scan was performed using a digital high-resolution PET/CT system (Biograph Vision 600, Siemens Healthineers, Knoxville, TN, USA), following the intravenous injection of 235 MBq of 18F-fluorodeoxyglucose (Gluscan, Advanced Accelerator Applications Molecular Imaging, Saint-Genis-Pouilly, France). The PET data were acquired using FlowMotion technology at a table speed of 0.7 mm/second (total acquisition duration = 8 min).

To ensure the reproducibility of patient positioning and facilitate accurate image registration, both 4D-CT and 4D-PET/CT scans were acquired using the same immobilization setup and respiratory monitoring system i.e., the RGSC system. The PET/CT data were retrospectively reconstructed into two sets: 1. 4D-PET/CT with 10 gates corresponding to the breathing cycle, and 2. static PET/CT.

The reconstruction parameters for both the static and 4D PET images aligned with the institutional protocols. A broader filter (4 mm vs. 2 mm) was applied to the 4D PET reconstruction to mitigate noise due to lower counting statistics in each of the 10 respiratory gates. In our institution, gating accuracy for both the imaging systems is routinely validated through periodic quality assurance procedures, which assess the correct respiratory cycle capture and reconstruction fidelity of all 10 respiratory phases.

### 2.3. Target Volume Segmentation

A custom Python 3.7 script was utilized to generate the Maximal Intensity Projection (MIP) of the 4D-PET image. Both the static PET/CT and 4D PET/CT (including the MIP) images were imported into Varian Eclipse 16.1 TPS and fused with those of planning 4D-CT. On each PET image, a 5 cm diameter spherical Region Of Interest (ROI) was delineated in the homogeneous uptake area of the liver. The noise level in the reconstructed image was defined as the standard deviation (SD) of SUV within this ROI. In order to improve target volume segmentation reproducibility, the Gross Tumor Volume (GTV) and the internal target volume (ITV) were segmented on the static and 4D-MIP PET images, respectively, using an SUV threshold that accounts for both the maximal SUV in the lesion and the noise level, as defined in Equation (1):

SUV_threshold_ = SD_liver_ + (SUV_max,lesion_ − SD_liver_)/2, (1)

This thresholding method based entirely on quantitative PET metrics was specifically selected to minimize intra-observer variability in target delineation.

**Figure 2:**
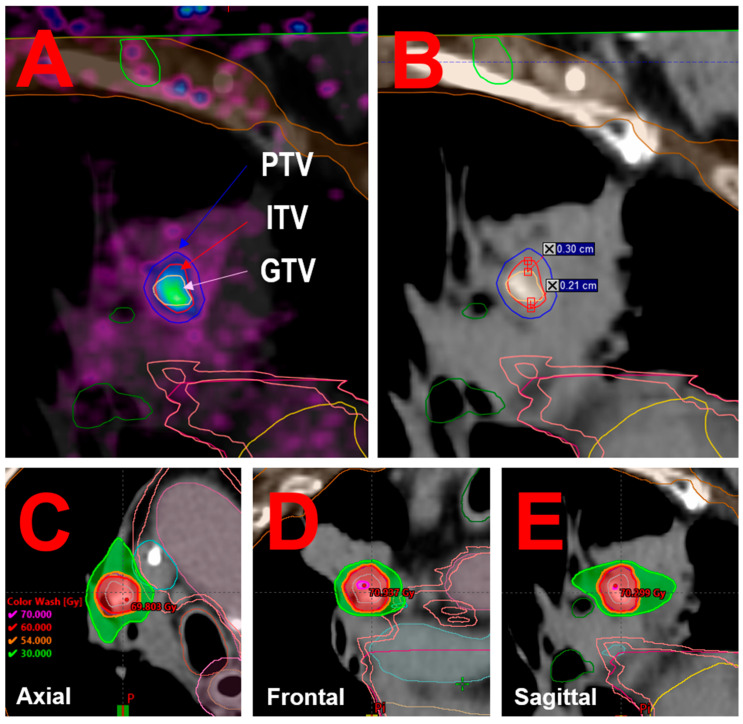
The sagittal views from planning 4D-MIP PET fused with planning CT (**A**) and planning CT alone, (**B**) illustrating the GTV delineated on the static PET, the ITV delineated on 4D-MIP PET, and the PTV. The images also highlight measurements of the inferior–superior discrepancies between the GTV and the ITV. The axial, coronal, and sagittal views from planning CT, showing the calculated dose distribution as isodose lines (**C**), (**D**) and (**E**) respectively).

Comparing static PET/CT and 4D-PET/CT is therefore equivalent to comparing the primary target volumes derived from each modality: GTV for static PET/CT and ITV for 4D-PET/CT (PET MIP). The segmented volumes were 0.5 cc for the GTV and 0.9 cc for the ITV. While no significant differences were observed in the lateral or anterior–posterior directions, the ITV extended more extensively in the craniocaudal direction. Specifically, the GTV spanned up to 8.0 mm, whereas the ITV reached 13.1 mm, with a maximum discrepancy of 5.1 mm between the two volumes (see Figure 2A,B).

The PTV was then defined by applying a uniform 3 mm isotropic margin to the ITV as per institutional protocol [26].

### 2.4. Treatment Planning and Delivery

The ITV was situated approximately 1 cm from the proximal bronchial tree, classifying the tumor as central per the LungTech trial criteria [8]. Consequently, the patient was prescribed a total dose of 60 Gy administered in eight fractions of 7.5 Gy each. According to the LungTech protocol, 95% of the PTV received at least the nominal fraction dose, and 99% of the PTV received a minimum of 54 Gy. The maximum dose within the PTV ranged from 66 to 78 Gy. The patient was treated every 2 days, except during the weekend.

Treatment planning was performed using Varian Eclipse 16.1 TPS. A volumetric modulated arc therapy plan was optimized and calculated with the AIP dataset using the Acuros XB algorithm (version 15.6.04), with a 1 mm dose calculation grid size and a dose to water reporting [27]. The plan consisted of two partial arcs rotating clockwise and counterclockwise from 181° to 0°, with collimator angles set to 45° and 315°, respectively. Dose distribution is illustrated in Figure 2C–E.

To evaluate the dosimetric impact of motion-inclusive imaging, two treatment plans were developed. The clinical plan was based on the ITV derived from the 4D-PET MIP image, expanded by 3 mm to form the PTV. A comparative plan was created using the GTV contoured on the static PET scan also expanded by 3 mm. Both the plans used the same prescription and optimization parameters and were normalized so that each plan-specific PTV coverage was 95% at 60 Gy. As previously described in Section 2.3, the 4D-PET-based ITV encompassed a larger motion envelope than the static PET-based GTV, particularly in the craniocaudal direction. This difference translated into a larger PTV (3.3 cc vs. 2.0 cc), and consequently improved coverage. The clinical plan achieved 95.0% PTV coverage with the prescribed dose, whereas the static PET-based plan would have covered only 61.7% of the 4D-PET-based PTV (see Table 1), highlighting the potential risk of underdosing when respiratory motion is not adequately accounted for.

Treatment was delivered using a Varian TrueBeam STx linear accelerator (Varian Medical Systems, Palo Alto, CA, USA) using 6 MV flattening filter-free beams. Before each fraction, patient positioning was verified using onboard kilovoltage orthogonal imaging, followed by a 4D Cone-Beam Computed Tomography (CBCT) to optimize soft-tissue alignment. Final positioning approval was given by a radiation oncologist prior to beam delivery during free breathing with an ITV strategy, and intrafraction patient motion tracking was conducted using combined surface optical/thermal imaging using the Brainlab ExacTrac Dynamic system (Brainlab AG, Munich, Germany).

### 2.5. Clinical Outcomes

**Figure 3:**
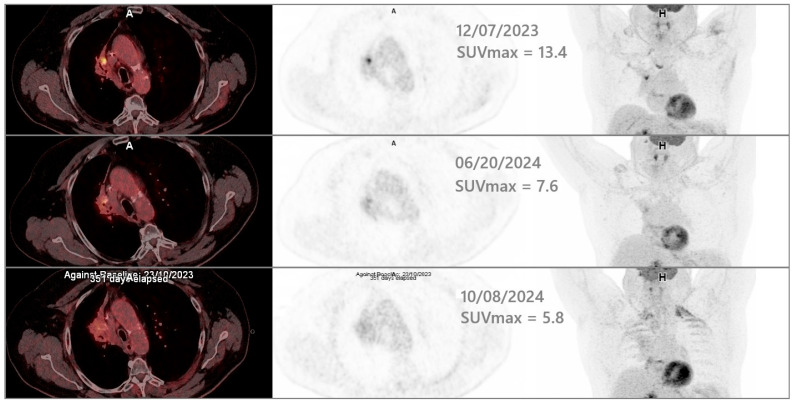
The axial and frontal MIP views from the successive time-points PET/CT. The first line shows the baseline PET/CT acquired before SBRT. The second line shows follow-up PET/CT conducted 3 months after the end of SBRT administration. The third line shows follow-up PET/CT conducted 7 months after the end of SBRT administration.

The SBRT was administered between 26 February and 14 March 2024. The patient exhibited excellent immediate tolerance, with no acute adverse effects, such as skin reactions, esophageal pain, dysphagia, increased dyspnea, and a cough. Follow-up PET/CT on 20 June 2024 demonstrated a reduction in the hypermetabolic focus (SUVmax 7.6 compared to 13.4 in December 2023). During a clinical evaluation on 1 July 2024, the patient reported stable dyspnea, the absence of bleeding, and no pain, indicating good acute tolerance to radiotherapy. Subsequent PET/CT on 8 October 2024 showed the further regression of the local hypermetabolism (SUVmax 5.8 compared to 7.6 in June 2024). Figure 3 shows regression between three time points: December 2023 (baseline), June 2024 (after SBRT administration), and October 2024 (follow-up). At one-year follow-up (25 February 2025), pulmonary function remained stable, and no late radiation-induced toxicity was reported.

## 3. Discussion

The use of 4D-PET/CT has gained increasing attention in lung radiotherapy, offering improved accuracy in tumor delineation by integrating both metabolic activity and respiratory motion. Conventional free-breathing PET/CT is prone to motion blurring and the underestimation of the tumor extent, particularly in the lower lobes or mobile lesions. In contrast, 4D-PET/CT enables motion-resolved imaging across the respiratory cycle, resulting in more accurate and reproducible target volumes. Several studies have shown that the GTVs defined using 4D-PET/CT are consistently larger and more anatomically representative than those obtained from static PET/CT [13,28].

These geometric improvements carry significant dosimetric implications. Siva et al. demonstrated that plans based on static PET volumes failed to adequately cover the motion-inclusive targets defined by 4D-PET/CT, risking a geographic miss and treatment underdosage [25]. Conversely, Zhang et al. showed that 4D-PET/CT could also help avoid overtreatment by excluding non-tumoral fibrosis and atelectasis from the target, thereby sparing healthy lung and cardiac tissues [24]. These benefits are particularly relevant in high-risk patients, where the therapeutic window is narrow and minimizing the collateral dose is critical.

In addition to enhancing target accuracy, 4D-PET/CT improves inter-observer consistency and delineation confidence, particularly in complex regions. Chirindel et al. found that for centrally located tumors, 4D-PET/CT significantly improved the agreement among clinicians and helped distinguish viable tumors from adjacent fibrotic or atelectatic tissue, scenarios where CT contrast alone may be insufficient [29]. This is especially valuable in recurrent disease where anatomy is altered by prior treatments.

Despite these advantages, the role of 4D-PET/CT in lung re-irradiation remains underexplored. To our knowledge, no prospective studies have specifically evaluated its use in retreatment scenarios. However, the case-based evidence, including the present report, suggests that 4D-PET/CT may be uniquely beneficial in differentiating recurrence from radiation-induced fibrosis and in minimizing the re-irradiated volume. CT-based delineation alone may be confounded by dense post-radiation changes, and even conventional PET may underestimate the lesion extent due to motion averaging and inflammatory uptake. Four-dimensional PET/CT overcomes these limitations by resolving motion and capturing metabolically active regions throughout the breathing cycle.

Recent multicenter analyses of lung re-irradiation, including those by John et al. and Sahin et al., underscore the importance of modern imaging tools such as PET/CT and 4D-CT for safe and effective SBRT retreatment [18,23]. However, none have yet systematically integrated 4D-PET/CT into the planning workflow. Our case illustrates that its use can enhance targeting accuracy, enabling tighter PTVs without compromising coverage, a crucial balance in anatomically constrained and previously irradiated lung tissue.

In our patient, the 67-year-old male with a centrally recurring squamous cell carcinoma embedded in post-radiation fibrosis, precise delineation was critical due to the tumor’s proximity to the bronchial tree and the surrounding fibrotic changes. As described in Section 2.3, the ITV delineated from 4D-PET/CT was nearly twice as large as the volume of the GTV from static PET (0.9 cc vs. 0.5 cc), with a craniocaudal discrepancy of 5.1 mm, exceeding our institutional PTV expansion margin of 3 mm [26]. To assess the dosimetric implications of this discrepancy, a theoretical treatment plan based on the static PET-derived GTV was generated. This plan covered only 61.7% of the clinical PTV with the prescribed dose compared to 95.0% in the plan based on the 4D-PET-derived ITV. These results clearly illustrate the risk of a geographic miss and treatment underdosage when respiratory motion is not adequately accounted for, particularly in mobile and fibrotic regions.

By contrast, the treatment plan guided by 4D-PET/CT ensured robust target coverage and allowed for the safe delivery of high-dose SBRT. The patient tolerated the treatment without any acute toxicity, including skin reactions, esophagitis, dysphagia, and increased dyspnea. The follow-up PET/CT scans demonstrated a sustained metabolic response, with the SUVmax decreasing from 13.4 in December 2023 to 5.8 in October 2024. At the one-year follow-up, pulmonary function remained stable, and no late radiation-induced toxicity was reported. These findings support the clinical utility and safety of 4D-PET/CT-guided re-irradiation. However, the absence of systematically collected quality-of-life data remains a limitation, underscoring the need for prospective studies that include patient-reported outcomes alongside imaging and dosimetric endpoints.

Although MRI has been proposed for distinguishing recurrence from fibrosis, its utility in thoracic imaging is constrained by respiratory or cardiac motion artifacts, low proton density in lung tissue, and challenges in post-radiation anatomy [30]. In contrast, 4D-PET/CT offers a robust combination of temporal and metabolic resolutions, making it more suitable for lesion characterization in fibrotic and moving lung regions. In this case, it was instrumental in differentiating the recurrent tumor from surrounding post-treatment changes, ensuring both accurate targeting and OAR sparing.

As this is a single case report, no statistical inference was performed. The volumetric and dosimetric data presented are descriptive and intended to illustrate the clinical utility of 4D-PET/CT in a high-risk and anatomically complex re-irradiation setting. The findings are not generalizable, but highlight the potential of this imaging modality to guide treatment in similarly challenging cases.

Due to the high-risk nature of thoracic re-irradiation, particularly near the central airway, the decision to proceed with SBRT in this case followed a rigorous multidisciplinary evaluation process. The indication was first reviewed by a general tumor board comprising radiation oncologists, medical oncologists, pulmonologists, radiologists, and nuclear medicine specialists. It was subsequently re-evaluated during treatment planning by a dedicated internal re-irradiation board, involving all the senior radiation oncologists of the department, medical physicists, and experienced radiation therapy technologists. A detailed risk–benefit discussion was held with the patient, who provided written informed consent for both the treatment and the publication of this case report. To mitigate the risks associated with prior irradiation and central tumor location, several safety measures were implemented: a moderate hypofractionation scheme (8 × 7.5 Gy), tight 3 mm PTV margins derived from motion-inclusive 4D imaging, daily image guidance using 4D CBCT, and real-time motion monitoring via an optical/thermal tracking system. This integrated approach underscores how careful planning and institutional safeguards can enable safe and effective treatment, even in anatomically and clinically challenging re-irradiation scenarios.

## 4. Conclusions

This case exemplifies the critical role of 4D-PET/CT in enhancing the precision of target volume delineation, particularly in complex scenarios involving tumor recurrence within post-irradiation fibrosis and proximity to critical structures. The observed discrepancies between the static PET (GTV) and 4D-MIP PET (ITV) volumes underscore the potential risks of underestimating tumor motion and extent when relying solely on static imaging. These findings advocate for the broader adoption of 4D-PET/CT in radiotherapy planning for thoracic malignancies, especially in highly complex clinical cases.

## Figures and Tables

**Table 1:** Target volumes and dose distributions from 4D PET/CT versus static PET/CT.

Imaging Modality Used for Target Volumes Contouring	4D PET/CT MIP	Static PET/CT
Primary target volume	ITV Volume = 0.9 cc Maximal dimension in infero superior axis = 13.1 mm	GTV Volume = 0.5 cc Maximal dimension in infero superior axis = 8.0 mm
Planning Target Volume (primary target volume + 3 mm isotropic margins)	Clinical PTV Volume = 3.3 cc Maximal dimension in infero superior axis = 19.1 mm	Theoretical PTV Volume = 2.0 cc Maximal dimension in infero superior axis = 14.0 mm
Clinical PTV coverage at the prescription dose of 60 Gy	95.0%	61.7%

## Data Availability

The data presented in this study are available on request from the corresponding author due to (specify the reason for the restriction).

## Abbreviations

The following abbreviations are used in this manuscript:

4D-CT	Four-Dimensional Computed Tomography
4D-PET/CT	Four-Dimensional Positron Emission Tomography/Computed Tomography
AIP	Average Intensity Projection
CBCT	Cone Beam Computed Tomography
GTV	Gross Tumor Volume
ITV	Internal Garget Volume
MIP	Maximum Intensity Projection
OAR	Organ At Risk
PTV	Planning Target Volume
RGSC	Respiratory Gating for SCanners
ROI	Region Of Interest
SBRT	Stereotactic Body Radiation Therapy
SD	Standard Deviation
SUV	Standardized Uptake Value
TPS	Treatment Planning System
